# Metabolome profiling by untargeted metabolomics and biomarker panel selection using machine-learning for patients in different stages of peripheral neuropathy induced by oxaliplatin

**DOI:** 10.3389/fonc.2025.1617207

**Published:** 2025-09-19

**Authors:** Yu-jiao Hua, Ying Zhang, Rui-Rong Wu, Juan Lv, Yan Zhang, Yan-yan Chen, Yong-juan Ding, Jing-hua Chen

**Affiliations:** ^1^ School of Life Sciences and Health Engineering, Jiangnan University, Wuxi, Jiangsu, China; ^2^ School of Chemical & Material Engineering, Jiangnan University, Wuxi, Jiangsu, China; ^3^ Department of Clinical Pharmacy, Affiliated Hospital of Jiangnan University, Wuxi, Jiangsu, China; ^4^ Department of Medical Oncology, Affiliated Hospital of Jiangnan University, Wuxi, Jiangsu, China; ^5^ Cancer Institute, Institute of Integrated Chinese and Western Medicine, Affiliated Hospital of Jiangnan University, Wuxi, Jiangsu, China

**Keywords:** oxaliplatin-induced peripheral neuropathy, biomarkers, untargeted metabolomics, machine-learning, colorectal cancer

## Abstract

**Background:**

Oxaliplatin-induced peripheral neuropathy (OIPN) poses a significant challenge for patients with colorectal tumor, often resulting in treatment interruption or discontinuation and subsequent treatment failure. Herein, a longitudinal untargeted metabolomic study to reveal the metabolomic profiles and biomarkers associated with the progression of OIPN.

**Methods:**

A prospective cohort of 129 colorectal cancer patients receiving oxaliplatin-based chemotherapy was stratified into four OIPN severity grades (Level 0-3). Serum samples underwent untargeted LC-MS/MS metabolomic analysis, detecting 521 metabolites. Multivariate statistical models and SHAP-guided random forest algorithms were employed to prioritize biomarkers. Machine learning validation included six classifiers assessed via ROC-AUC.

**Results:**

The cumulative dose of Oxaliplatin chemotherapy plays an important role in OIPN. At the same time, our findings implied that the occurrence of OIPN may be associated with the progression of the disease and the patients’ tumor markers (CEA, CA19-9, CA72-4), as well as immune response and inflammation (ANC, PLT), and metabolic and liver function abnormalities (GGT and UA) (P<0.05).Multivariate statistical analysis combined with SHAP-guided machine learning identified six biomarkers, including thiabendazole, 1-methylxanthine, imidazol-5-yl-pyruvate, 5-hydroxypentanoic acid, spermidine, and 4’-oxolividamine that consistently distinguished OIPN patients (Level 1-3) from non-OIPN controls (Level 0). Machine learning models, validated across six classifiers, demonstrated near-perfect discrimination for early-stage OIPN (AUC nearly 1). However, differentiation between intermediate OIPN grades (Level 1 vs 2, Level 1 vs 3, Level 2 vs 3) yielded lower predictive accuracy (AUC: 0.549–0.843), likely due to cohort size limitations and reliance on subjective sensory-based grading. Pathway enrichment analysis highlighted dysregulation in ABC transporters, central carbon metabolism in cancer, amino acid metabolism, and linoleic acid metabolism, suggesting potential roles in OIPN pathogenesis.

**Conclusions:**

These findings suggest that the selected biomarkers could serve as a foundation for the prediction and management of OIPN in colorectal cancer patients.

## 1 Introduction

In 2004, the U.S. Food and Drug Administration authorized the use of oxaliplatin, a platinum-based chemotherapy, for the management of metastatic colorectal cancer (mCRC) (1). Despite its efficacy, oxaliplatin’s side effect profile includes a significant issue known as oxaliplatin-induced peripheral neurotoxicity (OIPN), which can be a critical factor limiting the dosage and may necessitate a pause in therapy. OIPN affects over 85% of patients following treatment with oxaliplatin (2, 3). This condition is a notable adverse effect that can result in a reduction of the administered dose or the discontinuation of treatment altogether. A hallmark of OIPN is sensory peripheral neuropathy, which manifests as symptoms like dysesthesias, paresthesia, and sensory deficits, typically in a pattern resembling the distribution of a stocking or glove (4). These symptoms are often accompanied by neuropathic pain and, less commonly, involve motor and/or autonomic nerve damage. There are two primary forms of OIPN: an acute peripheral sensory and motor toxicity that often develops during or shortly after the drug infusion. This type of neuropathy tends to resolve quickly. In contrast, some patients may develop peripheral sensory neuropathy as a cumulative effect of the drug’s cumulative dose. This form of neuropathy is generally more persistent, with a gradual resolution after ceasing treatment (5).

Metabolomics, a branch of omics technologies, offers a thorough profiling of the internal metabolites within living organisms. This approach has demonstrated its significance across various domains, including aiding in the diagnosis of diseases, unraveling the complexities of disease processes, pinpointing potential drug targets, and tailoring therapeutic interventions to individual patients (6). In contrast to genomics and proteomics, which typically track changes over periods of days or weeks, metabolomics can offer a snapshot of alterations that occur within seconds or minutes following an event. Untargeted metabolomics, which involves the qualitative and quantitative analysis of all low-molecular-weight metabolites, has become a powerful tool for uncovering novel biomarkers and elucidating complex pathophysiological pathways (7). Our team conducted previous studies on biomarkers of OIPN caused by oxaliplatin and found that racemethionine, stearolic acid, 5-aminopentanoic acid, erythritol, aminoadipic acid, and all-trans-retinoic acid were pinpointed as promising biomarkers for OIPN (8). Nevertheless, OIPN is a progressive process, we have not focused on the pattern of metabolites in different stages of OIPN. Given the dynamic nature of metabolites, it is crucial to investigate the differences and alterations in metabolites throughout the OIPN progress and identify stable biomarkers associated with progression of OIPN.

In this study, we aimed to elucidate the metabolomic profiles and the patterns of metabolite changes of OIPN progression using traditional statistical and machine learning methods. Identification of stable biomarkers associated with the progression of OIPN would enhance our understanding of the mechanisms of OIPN, also provided new insights and targets for the prevention and treatment of OIPN.

## 2 Materials and methods

### 2.1 Study population and data collection

This study was based on an ongoing prospective study conducted in the Affiliated Hospital of Jiangnan University. A total of 129 colorectal cancer patient receiving oxaliplatin chemotherapy twice were enrolled from August 2022 and July 2023. The criteria for patient selection are as follows: 1) histopathological confirmation of colorectal cancer diagnosis; 2) TNM staging ranging from I to IV; 3) chemotherapy involving oxaliplatin-containing regimens; 4) good general condition; 5) Karnofsky Performance Status (KPS) score greater than 60; 6) absence of other diseases causing peripheral neuropathy, such as diabetes; 7) no current use of medications affecting peripheral nerves; 8) age between 18 and 85 years, irrespective of gender; 9) PS score ≤ 2 points; 10)) expected survival period of more than 3 months; 11) normal liver, kidney, heart, bone marrow, and other functionalities; 12) patients with intact consciousness and the ability to clearly articulate their physical sensations. The study was conducted according to the guidelines under the Declaration of Helsinki and approved by Ethics Committee of the Affiliated Hospital of Jiangnan University (LS2022080). All the participants signed consent forms.

The OIPN was graded by physicians according to the National Cancer Institute (NCI) Common Adverse Reaction Evaluation Criteria (NCI-CTCAE V3.0) for grading peripheral nerve injury. OIPN can be classified into four severity levels: Level 0: patients with no OIPN; Level I: involves the disappearance of deep tendon reflexes or sensory abnormalities that do not impede physical function, manifesting as asymptomatic or detectable solely through examination; Level II: encompasses mild sensory changes or abnormalities (including needle-pricking sensations) that affect physical performance but do not disrupt daily life; Level III: presents with more severe abnormal sensory changes, requiring assistive devices such as canes or wheelchairs for mobility; Level IV: represents a disability or life-threatening condition. The demographic data including white blood cells (WBC), eosinophil (EOS), lymphocyte (LYM), alanine aminotransferase (ALT), aspartate aminotransferase (AST), total bilirubin (TBIL), direct bilirubin (DBIL), tumor markers, oxaliplatin dosage, and concomitant medication were collected by reviewing electronic medical records.

### 2.2 Sample pretreatment

A cohort of 129 serum samples, stored at -80 °C, underwent preparation for untargeted metabolomic analysis. Samples were thawed on ice and vortexed for 1 minute. Subsequently, 400 µL of methanol was dispensed into a 96-well plate, followed by the addition of 100 µL aliquots of each serum sample. The mixtures were subjected to vigorous vortex mixing for 5 minutes to ensure homogeneity. Sample plates were then transferred to an A200 pressurized nitrogen evaporator, where they underwent complete solvent evaporation under a controlled nitrogen stream for 10 minutes. The dried residues were reconstituted with 150 µL of a 4 ppm 2-chlorophenylalanine solution (prepared in 80% methanol) (CAS 103616-89-3; Aladdin Reagent Co.), followed by 5 minutes of vortex mixing to ensure complete dissolution. Finally, the reconstituted samples were membrane-sealed in preparation for subsequent LC-MS analysis.

### 2.3 LC-MS/MS analysis

The LC analysis was performed on a Vanquish UHPLC System (Thermo Fisher Scientific, USA). Chromatography was carried out with an ACQUITY UPLC ^®^ HSS T3 (2.1×100 mm, 1.8 µm) (Waters, Milford, MA, USA). For LC-ESI (+)-MS analysis, the mobile phases consisted of (B2) 0.1% formic acid in acetonitrile (v/v) and (A2) 0.1% formic acid in water (v/v). Separation was conducted under the following gradient: 0~1 min, 8% B2; 1~8 min, 8%~98% B2; 8~10 min, 98% B2; 10~10.1 min, 98%~8% B2; 10.1~12 min, 8% B2. For LC-ESI (-)-MS analysis, the analytes was carried out with (B3) acetonitrile and (A3) ammonium formate (5mM). Separation was conducted under the following gradient: 0~1 min, 8% B3; 1~8 min, 8%~98% B3; 8~10 min, 98% B3; 10~10.1 min, 98%~8% B3; 10.1~12 min, 8% B3. Mass spectrometric detection of metabolites was performed on Orbitrap Exploris 120 (Thermo Fisher Scientific, USA) with ESI ion source. Simultaneous MS1 and MS/MS acquisition was used. The parameters were as follows: sheath gas pressure, 40 arb; aux gas flow, 10 arb; spray voltage, 3.50 kV and -2.50 kV for ESI(+) and ESI(-), respectively; capillary temperature, 325 °C; MS1 range, m/z 100-1000; MS1 resolving power, 60000 FWHM; number of data dependant scans per cycle, 4; MS/MS resolving power, 15000 FWHM; normalized collision energy, 30%; dynamic exclusion time, automatic.

### 2.4 Data processing

The raw data were firstly converted to mzXML format by MSConvert in ProteoWizard software package (v3.0.8789) (9) and processed using R XCMS (v3.12.0) for feature detection (10), retention time correction and alignment. Key parameters settings were set as follows: ppm=15, peakwidth=c (5, 30), mzdiff=0.01, method=centWave. The batch effect was then eliminated by correcting the data based on QC samples. Metabolites with RSD > 30% in QC samples were filtered and then used for subsequent data analysis. The metabolites were identified by accuracy mass and MS/MS data which were matched with HMDB, massbank, KEGG, LipidMaps, mzcloud and the metabolite database build by Panomix Biomedical Tech Co., Ltd. (Shuzhou, China). The molecular weight of metabolites was determined according to the m/z (mass-to-charge ratio) of parent ions in MS data. Molecular formula was predicted by ppm (parts per million) and adduct ion, and then matched with the database to realize MS identification of metabolites. At the same time, the MS/MS data from quantitative table of MS/MS data, were matched with the fragment ions and other information of each metabolite in the database, so as to realize the MS/MS identification of metabolites.

### 2.5 Statistical analysis

Two different multivariate statistical analysis models, unsupervised and supervised, were applied to discriminate the groups, including pareto-scaled principal component analysis (PCA) and orthogonal partial least-squares discriminant analysis (OPLS-DA) by R ropls (v1.22.0) package (11). Furthermore, permutation test (200 times) and CV-ANOVA were performed to validate the generated models. The statistical significance of P-value was obtained by statistical test between groups. The variable importance in the projection (VIP) value of each variable in the OPLS-DA model was calculated to indicate its contribution to the classification. Finally, combined with P-value, VIP, and fold change (FC) (multiple of difference between groups) to screen biomarker metabolites. By default, when P value < 0.05 and VIP value > 1, metabolites were considered to have significant differential expression. Differential expressed metabolites (DEMs) were subjected to pathway analysis by MetaboAnalyst (12), which combines results from powerful pathway enrichment analysis with the pathway topology analysis. The identified metabolites in metabolomics were then mapped to the KEGG pathway for biological interpretation of higher-level systemic functions. The metabolites and corresponding pathways were visualized using KEGG Mapper tool.

### 2.6 Machine learning method for biomarker selection

Traditional statistical analyses do not consider interactive or modifying effects among different metabolites, which may lead to false positive results (13). SHAP, a cutting-edge technique for enhancing the interpretability of tree-based models, employs a game-theoretic approach to combine the local effects of each feature, thereby elucidating the model’s functioning across the entire dataset. This method is regarded as superior to other global approximation techniques. The SHAP algorithm not only quantifies the importance of features within the model but also delves into the specific influence of each feature on individual predictions (14). Therefore, we subsequently employed SHAP analysis workflow to identify biomarkers stably associated with OIPN.

Through the analysis of the random forest model, we have derived the Shapley values for every individual DEM. Following this, we have meticulously chosen the 20 DEMs with the most substantial Shapley values for inclusion in predictive models. These selected DEMs are now being applied across a spectrum of six machine learning algorithms (including K-Nearest Neighbors, Random Forest, Support Vector Machines, Gaussian Naive Bayes, Logistic Regression, and Decision Trees). To evaluate their predictive capabilities, we have also generated ROC curves, providing a graphical representation of the performance of each method. Data analysis was performed with the statistical software package R (v4.4.1).

## 3 Results

### 3.1 Demographic and clinical characteristics with OIPN

**Figure 1:**
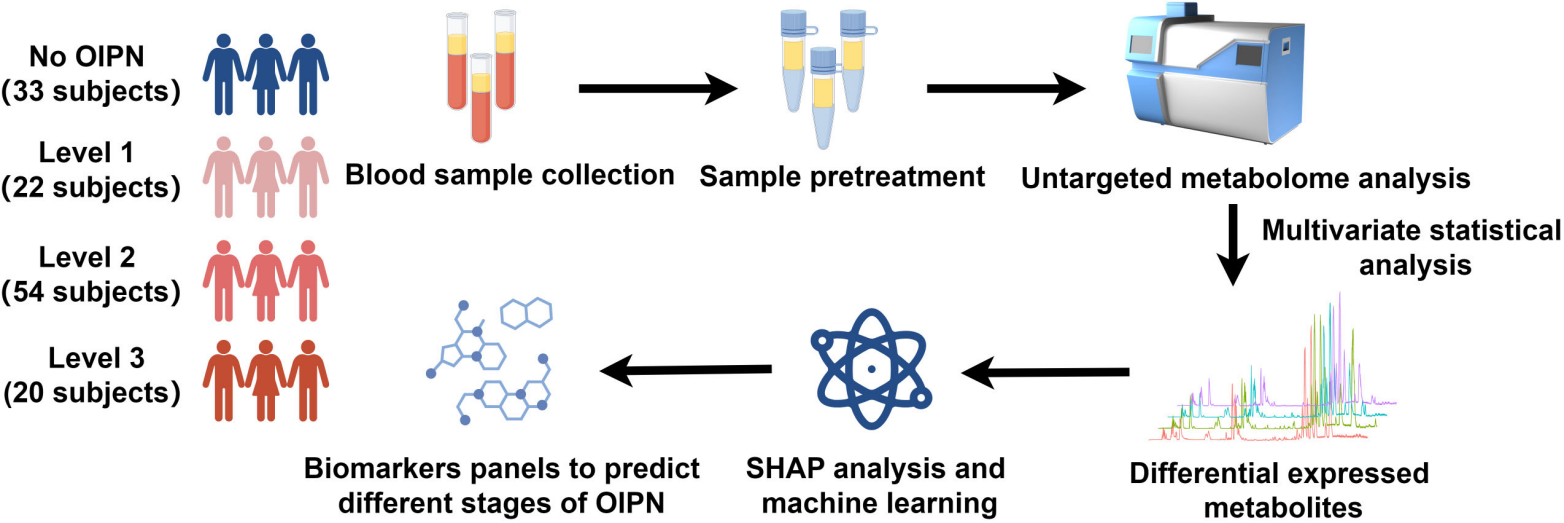
Schematic diagram of the study design. 33 participants with no OIPN, 22 participants with Level 1 OIPN, 54 participants with Level 2 OIPN, and 20 participants with Level 3 OIPN were used to perform untargeted metabolomics analysis to profile metabolic alterations in metabolic network analysis. SHAP analysis and six machine learning were employed to select potential metabolite biomarkers to predict the different stages of OPIN.

To profile the metabolic features in the serum of participants with different stages of OIPN, we performed a serum metabolomics study in a cohort of 33 participants with no OIPN (Level 0), 22 with Level 1 OIPN, 54 with Level 2 OIPN, and 20 with Level 3 OIPN (**Figure 1**). Detailed clinical parameters across the four OIPN severity groups are presented in **Table 1**. Specifically, the cumulative oxaliplatin (L-OHP) dose was 444.85±327.78 mg in Level 0, 448.86±268.83 mg in Level 1, 584.76±303.52 mg in Level 2, and 604±338.07 mg in Level 3, with significantly higher doses in Level 2 and Level 3 compared to Level 0 (p=0.034 and p=0.038, respectively), indicating a dose-dependent association with OIPN progression. On the other hand, across all OIPN severity levels (Level 0 to Level 3), the proportion of male patients was consistently higher than that of female patients. With respect to cancer staging, the majority of patients were classified as having stage III or IV colorectal cancer. Regarding treatment regimens, two main oxaliplatin-based chemotherapy protocols were administered: XELOX (capecitabine plus oxaliplatin) and FOLFOX (fluorouracil, leucovorin, plus oxaliplatin), with the XELOX regimen accounting for the larger proportion across all OIPN severity levels.

**Table 1:** Demographic and clinical characteristics of participants.

	Level 0 (n=33)	Level 1 (n=22)	Level 2 (n=54)	Level 3 (n=20)	p-value					
					level0 vs level1	level0 vs level2	level0 vs level3	level1 vs level2	level1 vs level3	level2 vs level3
Age (year)	61.27±8.73	62.91±7.79	59.78±8.95	64.75±9.37	0.50	0.45	0.17	0.16	0.50	0.034
Female (n, %)	11 (33.33%)	6 (27.28%)	12 (22.22%)	7 (35%)						
Cancer stage (I, II, III, IV) (n)	1, 2, 7, 11 (3.03%, 6.06%, 21.21%, 33.33%)	0, 2, 11, 9 (0%. 9.09%, 50%, 40.91%)	1, 17, 18, 18 (18.52%, 31.48%, 33.33%, 33.33%)	0, 7, 9, 14 (0%, 35%, 45%, 70%)						
Treatment type (XELOX,FOLFOX) (n, %)	22 (66.67%), 11 (33.33%)	13 (59.09%), 9 (40.91%)	38 (70.37%), 16 (29.63%)	18 (90%), 2 (10%)						
L-OHP dose, mg	444.85±327.78	448.86±268.83	584.76±303.52	604±338.07	0.91	0.034	0.038	0.083	0.071	0.65
WBC, 10^9^/L	5.75±1.20	5.85±2.24	5.21±2.62	4.33±1.22	0.99	0.69	0.12	0.67	0.13	0.44
ANC, 10^9^/L	3.61±1.72	3.38±1.71	3.11±2.42	2.15±0.90	0.68	0.26	0.010	0.59	0.044	0.061
ALC, 10^9^/L	1.56±0.45	1.89±1.43	1.47±0.41	1.63±0.47	0.099	0.56	0.74	0.022	0.24	0.40
EOS,10^9^/L	0.12±0.10	0.14±0.085	0.14±0.11	0.13±0.09	0.54	0.36	0.84	0.89	0.72	0.58
ABC,10^9^/L	0.015±0.036	0.032±0.048	0.011±0.032	0.0050±0.02	0.086	0.60	0.31	0.021	0.014	0.51
RBC, 10¹²/L	3.95±0.7404	4.11±0.52	3.79±0.77	3.79±0.52	0.40	0.30	0.41	0.071	0.14	0.99
HGB, g/L	115.67±27.00	123.77±15.87	116.25±25.21	121.5±13.98	0.20	0.91	0.37	0.20	0.75	0.38
PLT,10^9^/L	213.39±76.22	188.91±72.39	171.54±71.12	134.90±48.27	0.21	0.0080	0	0.33	0.013	0.047
AFP, ng/mL	3.62±3.27	20.22±69.93	2.62±1.39	2.85±1.16	0.039	0.88	0.92	0.018	0.053	0.97
CEA, ng/mL	146.14±243.13	72.62±149.56	15.16±27.59	31.21±81.01	0.084	0	0.010	0.14	0.38	0.69
CA19-9, U/mL	312.62±469.038	35.23±97.16	53.80±92.19	43.18±78.14	0	0	0.0010	0.79	0.93	0.89
CA72-4, U/mL	33.66±68.76	6.57±5.38	9.39±16.045	5.65±8.63	0.02	0.012	0.023	0.79	0.94	0.74
CA-125, U/mL	21.36±26.95	22.71±71.69	13.51±12.83	11.66±13.75	0.89	0.31	0.32	0.29	0.30	0.84
TBIL, μmol/L	13.36±6.45	12.98±4.24	12.27±6.073	13.92±9.93	0.84	0.46	0.76	0.67	0.65	0.34
DBIL, μmol/L	2.75±1.76	2.46±0.84	2.82±3.48	2.36±1.19	0.68	0.80	0.59	0.57	0.90	0.48
ALB, g/L	38.015±4.61	39.95±2.87	38.21±3.87	37.89±2.25	0.063	0.82	0.90	0.069	0.077	0.74
PA, mg/L	233.91±71.66	254.81±43.23	219.27±71.94	210.96±41.02	0.24	0.30	0.21	0.030	0.028	0.62
ALT, U/L	26.97±16.95	38.41±46.63	30.13±23.96	21.15±9.73	0.12	0.59	0.44	0.22	0.036	0.20
GDH, U/L	10.35±13.89	46.38±188.51	36.92±202.98	5.11±2.99	0.33	0.43	0.90	0.81	0.38	0.43
GGT, U/L	85.39±110.37	38.2±28.00	58.87±70.11	28.4±11.82	0.021	0.10	0.0070	0.27	0.66	0.11
CHE, U/L	6014.27±1934.31	7269.45±1513.00	6251.35±2085.66	6879.75±1362.088	0.016	0.57	0.10	0.033	0.50	0.20
Fe, μmol/L	10.86±6.43	14.66±5.84	12.47±7.093	13.83±6.23	0.039	0.27	0.12	0.19	0.69	0.43
GLU, mmol/L	4.93±0.68	5.49±1.30	5.21±1.036	4.91±0.51	0.033	0.18	0.92	0.24	0.047	0.22
Cr, μmol/L	64.50±19.08	66.54±13.93	66.74±18.17	61.81±7.69	0.66	0.54	0.57	0.96	0.38	0.26
UA, μmol/L	347.55±100.62	387.01±84.70	336.52±88.05	317.81±58.83	0.10	0.57	0.23	0.024	0.011	0.42
RBP, mg/L	36.55±13.076	40.57±7.94	34.40±11.35	33.28±8.42	0.19	0.38	0.31	0.029	0.038	0.71
TC, mmol/L	5.11±1.079	4.63±0.71	4.74±1.075	4.86±0.52	0.085	0.094	0.37	0.68	0.46	0.64
TG, mmol/L	2.00±1.20	1.94±1.32	1.78±1.013	1.59±0.72	0.84	0.39	0.20	0.60	0.32	0.51
HDL-C, mmol/L	1.19±0.34	1.13±0.26	1.12±0.32	1.21±0.24	0.51	0.33	0.84	0.90	0.45	0.32
LDL-C, mmol/L	3.21±0.62	2.90±0.50	3.072±0.81	3.056±0.44	0.13	0.40	0.48	0.35	0.51	0.94

Values are given as mean ± SD. WBC, White Blood Cell; ANC, Absolute Neutrophil Count; ALC, Absolute Lymphocyte Count; EOS, Eosinophil Absolute Count; ABC, Absolute Basophil Count; RBC, Red Blood Cell Count; HGB, Hemoglobin; PLT, Total Platelet Count; AFP, Alpha fetoprotein; CEA, Carcinoembryonic Antigen; CA19-9, Carbohydrate Antigen 19-9; CA72-4, Carbohydrate Antigen 72-4; CA-125, Carbohydrate Antigen 125; TBIL, Total Bilirubin; DBIL, Direct Bilirubi; ALB, Albumin; PA, Prealbumin; ALT, Alanine Aminotransferase; GDH, Glutamate Dehydrogenase; GGT, Gamma-Glutamyl Transpeptidase; CHE, Cholinesterase; Fe, Iron; GLU, Glucose; Cr, Creatinine; UA, Uric Acid; RBP, Retinol-Binding Protein; TC, Total Cholesterol; TG, Triglyceride; HDL-C, High - density Lipoprotein Cholesterol; LDL-C, Low - density Lipoprotein Cholesterol.

For hematological indices, white blood cell (WBC) counts were 5.75±1.99×10^9^/L (Level 0), 5.85±2.24×10^9^/L (Level 1), 5.21±2.62×10^9^/L (Level 2), and 4.33±1.22×10^9^/L (Level 3), with no significant intergroup differences. Absolute lymphocyte counts (ALC) were 1.56±0.45×10^9^/L (Level 0), 1.89±1.43×10^9^/L (Level 1), 1.47±0.41×10^9^/L (Level 2), and 1.63±0.47×10^9^/L (Level 3), with a significant difference between Level 1 and Level 2 (p=0.022). Eosinophil counts (EOS) exhibited no significant differences across groups: 0.12±0.10×10^9^/L (Level 0), 0.14±0.085×10^9^/L (Level 1), 0.14±0.11×10^9^/L (Level 2), and 0.13±0.09×10^9^/L (Level 3). Red blood cell (RBC) counts (3.95±0.74×10¹²/L, 4.11±0.52×10¹²/L, 3.79±0.77×10¹²/L, 3.79±0.52×10¹²/L for Levels 0–3) and hemoglobin (HGB) levels (115.67±27.00 g/L, 123.77±15.87 g/L, 116.25±25.21 g/L, 121.5±13.98 g/L) also did not differ significantly between groups.

Regarding liver function and metabolic indices, total bilirubin (TBIL: 13.36±6.45 μmol/L, 12.98±4.24 μmol/L, 12.27±6.07 μmol/L, 13.92±9.93 μmol/L), direct bilirubin (DBIL: 2.75±1.76 μmol/L, 2.46±0.84 μmol/L, 2.82±3.48 μmol/L, 2.36±1.19 μmol/L), albumin (ALB: 38.015±4.61 g/L, 39.95±2.87 g/L, 38.21±3.87 g/L, 37.89±2.25 g/L), and glutamate dehydrogenase (GDH: 10.35±13.89 U/L, 46.38±188.51 U/L, 36.92±202.98 U/L, 5.11±2.99 U/L) showed no significant intergroup differences. Total cholesterol (TC: 5.11±1.08 mmol/L, 4.63±0.71 mmol/L, 4.74±1.08 mmol/L, 4.86±0.52 mmol/L) also did not differ significantly across groups.

Tumor markers exhibited notable variations: carcinoembryonic antigen (CEA) levels were 146.14±243.13 ng/mL (Level 0), 72.62±149.56 ng/mL (Level 1), 15.16±27.59 ng/mL (Level 2), and 31.21±81.01 ng/mL (Level 3), with Level 0 showing significantly higher levels than Level 2 (p<0.001) and Level 3 (p=0.010). Carbohydrate antigen 19-9 (CA19-9) was 312.62±469.04 U/mL (Level 0), 35.23±97.16 U/mL (Level 1), 53.80±92.19 U/mL (Level 2), and 43.18±78.14 U/mL (Level 3), with Level 0 significantly higher than all other groups (p<0.001). Carbohydrate antigen 72-4 (CA72-4) was 33.66±68.76 U/mL (Level 0), 6.57±5.38 U/mL (Level 1), 9.39±16.05 U/mL (Level 2), and 5.65±8.63 U/mL (Level 3), with Level 0 significantly higher than Levels 1–3 (p=0.020, 0.012, 0.023).

Immune and inflammatory indices showed significant differences: absolute neutrophil counts (ANC) were 3.61±1.72×10^9^/L (Level 0), 3.38±1.71×10^9^/L (Level 1), 3.11±2.42×10^9^/L (Level 2), and 2.15±0.90×10^9^/L (Level 3), with Level 0 higher than Level 3 (p=0.010) and Level 1 higher than Level 3 (p=0.044). Platelet (PLT) counts were 213.39±76.22×10^9^/L (Level 0), 188.91±72.39×10^9^/L (Level 1), 171.54±71.12×10^9^/L (Level 2), and 134.90±48.27×10^9^/L (Level 3), with progressive decreases showing significant differences between Level 0 vs Level 2 (p=0.008), Level 0 vs Level 3 (p<0.001), Level 1 vs Level 3 (p=0.013), and Level 2 vs Level 3 (p=0.047). Uric acid (UA) levels were 347.55±100.62 μmol/L (Level 0), 387.01±84.70 μmol/L (Level 1), 336.52±88.05 μmol/L (Level 2), and 317.81±58.83 μmol/L (Level 3), with Level 1 higher than Level 2 (p=0.024) and Level 3 (p=0.011). All p-values <0.05 indicate statistically significant differences between the corresponding groups (**Table 1**).

### 3.2 Detection of endogenous metabolites in plasma

**Figure 2:**
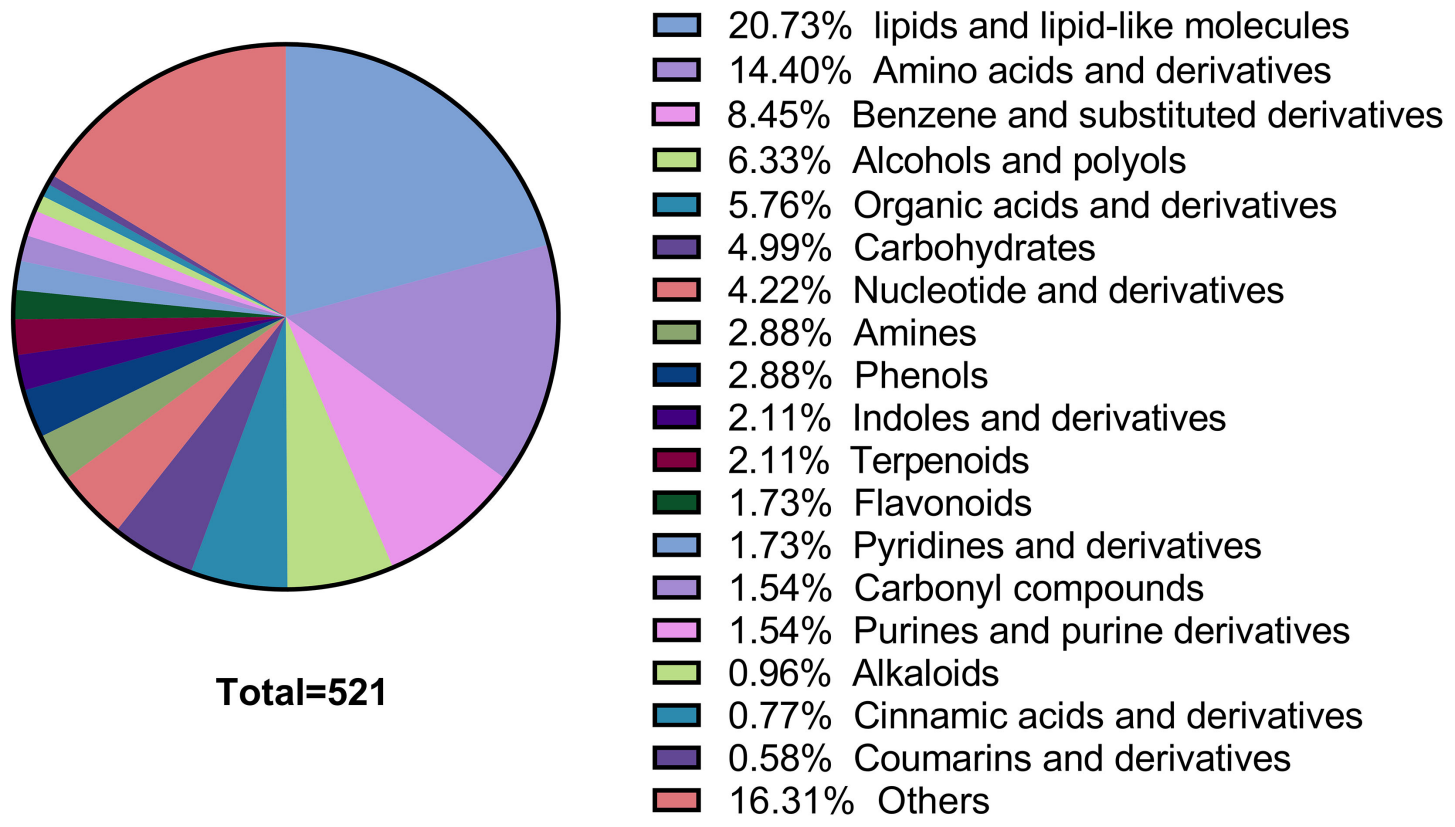
The number proportion of identified metabolites in each chemical classification.

The total ion chromatogram (TIC) profiles exhibited substantial overlap between technical replicates, with consistent retention times and peak intensities across analyses, demonstrating high reproducibility and stability of the chromatographic signals throughout the analytical sequence (**Supplementary Figures S1A, B**). Concurrently, the tight clustering of quality control (QC) sample data points observed in the principal component analysis (PCA) score plots generated in both positive (**Supplementary Figure S1C**) and negative (**Supplementary Figure S1D**) ionization modes verifies stable instrumental performance and good analytical reproducibility throughout the experimental process, thereby ensuring the reliability of subsequent biological differences identified based on this dataset. LC-MS/MS-based metabolomic profiling identified 521 distinct metabolites, comprising 315 compounds in positive ionization mode and 206 in negative mode. These detected metabolites were categorized into 22 distinct biochemical classes based on their chemical taxonomy, with proportional distribution across categories illustrated in **Figure 2**. As shown in **Figure 2**, lipids and lipid-like molecules, which constitute the largest proportion at 20.73%, primarily include Fatty acids and conjugates lipids, and Steroids and steroid derivatives (e.g., dodecanoic acid, stearic acid, linoleic acid), supporting our claim of their predominance. Amino acids and derivatives account for 14.4%, encompassing compounds such as L-phenylalanine, 1-methylhistidine, and L-tyrosine. Benzene and substituted derivatives make up 8.45%, including benzaldehyde, methoxamine, and neostigmine, while alcohols and polyols represent 6.33% with examples like dihydrocortisol, smilagenin, and hecogenin. Organic acids and derivatives constitute 5.76%, featuring methylmalonic acid, 12-hydroxydodecanoic acid, and isocitric acid, and carbohydrates account for 4.99%, including D-mannose, mannitol, and fructose 1,6-bisphosphate. Nucleotide and derivatives make up 4.22%, with compounds such as uridine, deoxycytidine, and thymidine; Amines and phenols each account for 2.88%, including spermine, anandamide, tryptophanamide (amines) and m-cresol, chavicol, gingerol (phenols). Indoles and derivatives (2.11%, e.g., indole, indole-3-acetate) and terpenoids (2.11%, e.g., lanosterin, zingiberene) follow, while flavonoids (1.73%, e.g., quercetin, formononetin) and pyridines and derivatives (1.73%, e.g., pyridoxine, pirbuterol) are also present. Carbonyl compounds (1.54%, e.g., hydroxykynurenine) and purines and purine derivatives (1.54%, e.g., paraxanthine) are included, along with alkaloids (0.96%, e.g., anabasine), cinnamic acids and derivatives (0.77%, e.g., caffeic acid), and coumarins and derivatives (0.58%, e.g., ostruthin). The “others” category, comprising 16.31%, includes compounds such as zonisamide, bilirubin, and biotin, which do not fit into the aforementioned categories due to their unique structures or functions.

### 3.3 Identification of DEMs and its functional analysis in different stages of OIPN groups

**Figure 3:**
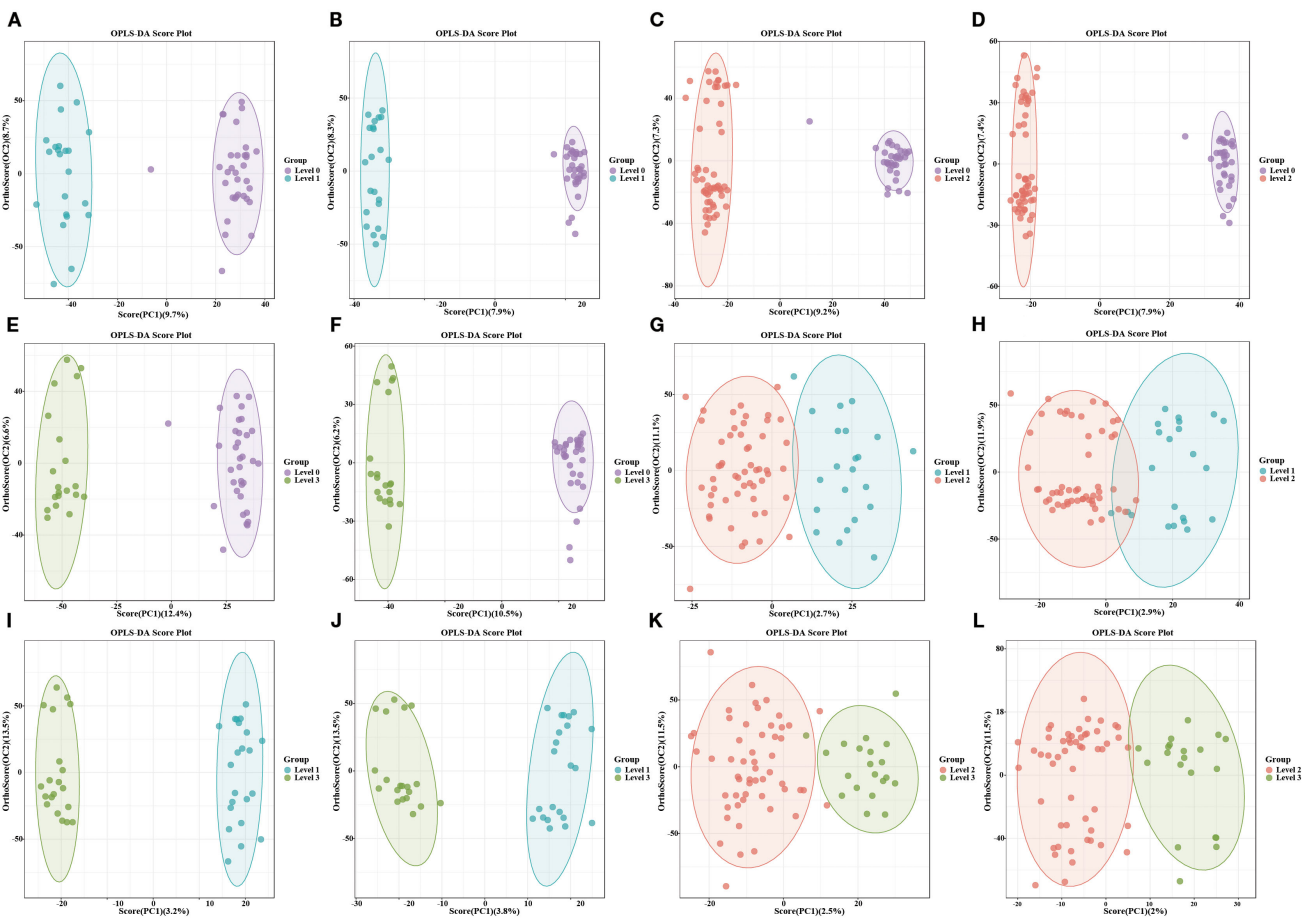
Distinct separations in metabolic profiles using OPLS-DA. **(A, C, E, G, I, K)**: OPLS-DA model in the Level 1vs Level 0, Level 2vs Level 0, Level 3vs Level 0, Level 2vs Level 1, Level 3vs Level 1, and Level 3vs Level 2 in the positive mode. **(B, D, F, H, J, L)**: OPLS-DA model in the Level 1vs Level 0, Level 2vs Level 0, Level 3vs Level 0, Level 2vs Level 1, Level 3vs Level 1, and Level 3vs Level 2 in the negative mode.

PCA initially displayed partial separations in both positive and negative modes in different stages of OIPN groups (**Supplementary Figure S2**). Furthermore, the OPLS-DA plots illustrated distinct separations in metabolic profiles in different stages of OIPN groups, which can be well distinguished in both positive and negative modes (**Figure 3**). The 200-permulation test of LC-MS data was shown in **Supplementary Figure S3**. All the values were lower than their corresponding original ones and the intercepted value of Q^2^ in the vertical axis was below 0.5 (15), suggesting the model was not overfitted. Therefore, the model produced goodness of prediction in different stages of OIPN groups.

**Figure 4:**
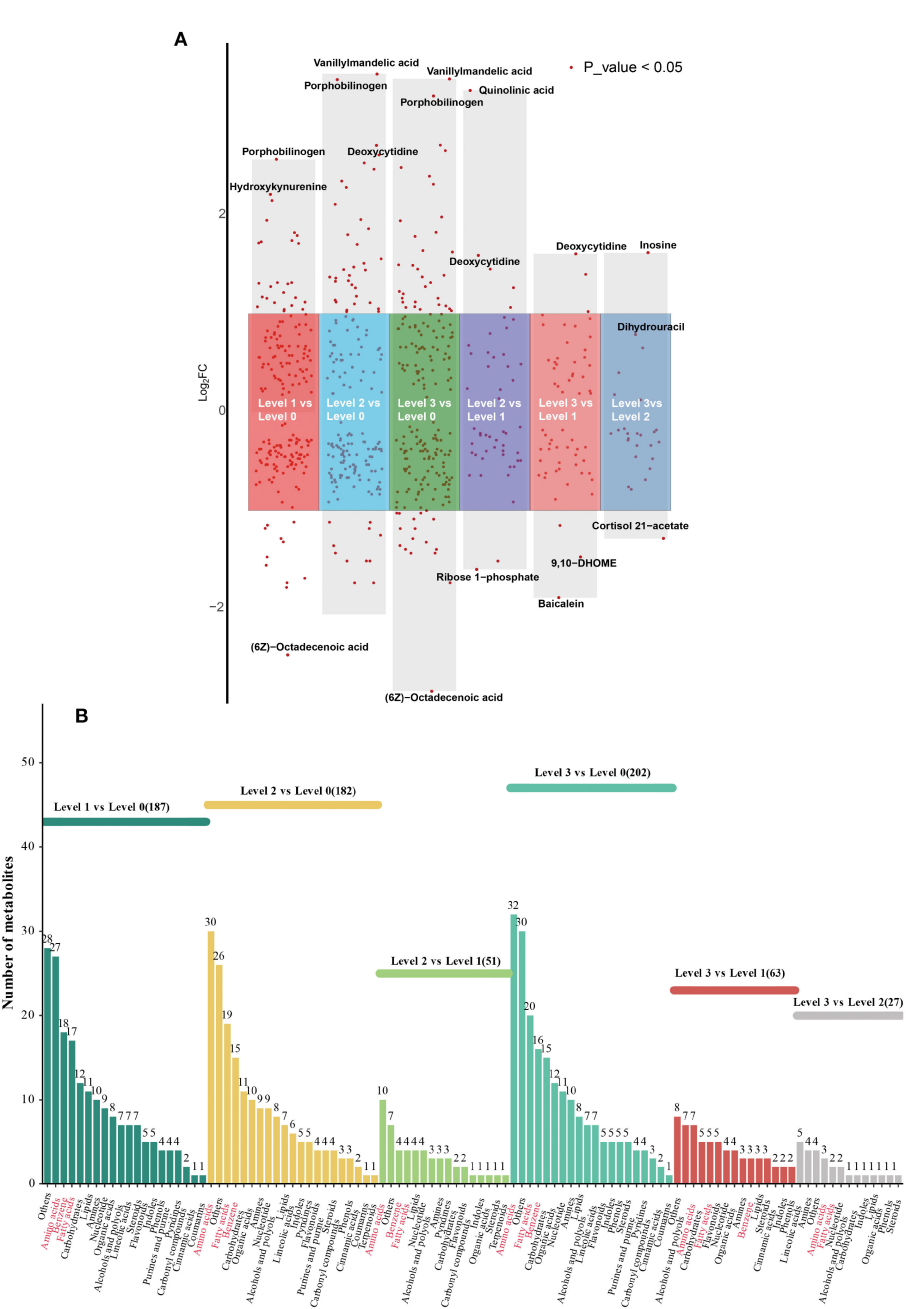
**(A)** Multi-group volcano map. DEMs: meet P value < 0.05 and VIP value > 1 are shown in red. **(B)** The classification of DEMs among six different groups.

To further explore the differential metabolite molecules with biological significance, we screened DEMs based on both VIP>1 and P-value < 0.05 in OPLS-DA. Specifically, we observed 187 DEMs in Level 1 vs Level 0 (including 97 up-regulated and 90 down-regulated), 182 in Level 2 vs Level 0 (including 78 up-regulated and 104 down-regulated), 202 in Level 3 vs Level 0 (including 99 up-regulated and 103down-regulated), 51 in Level 2 vs Level 1 (including 21 up-regulated and 30 down-regulated), 63 in Level 3 vs Level 1 (including 31 up-regulated and 32 down-regulated), and 27 in Level 3 vs Level 2 (including 7 up-regulated and 20 down-regulated) comparisons (**Figure 4A**). The top three most significantly regulated metabolites in each comparison group, ranked by absolute log_2_FC values, are explicitly labeled. Positive log_2_FC values denote upregulation, while negative values indicate downregulation. The complete dataset of DEMs with corresponding annotation details and quantitative measurements is provided in **Supplementary Table S1**. The result of screening for DEMs was visualized in histogram, with different colors to distinguish the classification of DEMs in different groups, which indicated that the DEMs across all groups were primarily concentrated in the categories of amino acids and derivatives, benzene and substituted derivatives, and fatty acids and conjugates (**Figure 4B**).

Enriched pathway analysis was performed for the screened metabolites by using KEGG database, related pathways could be classified into ABC transporters, central carbon metabolism in cancer, amino acid metabolism (including D-amino acid metabolism, arginine biosynthesis, arginine and proline metabolism), linoleic acid metabolism (**Supplementary Figure S4**).

### 3.4 Identification of biomarkers using machine learning method

**Figure 5:**
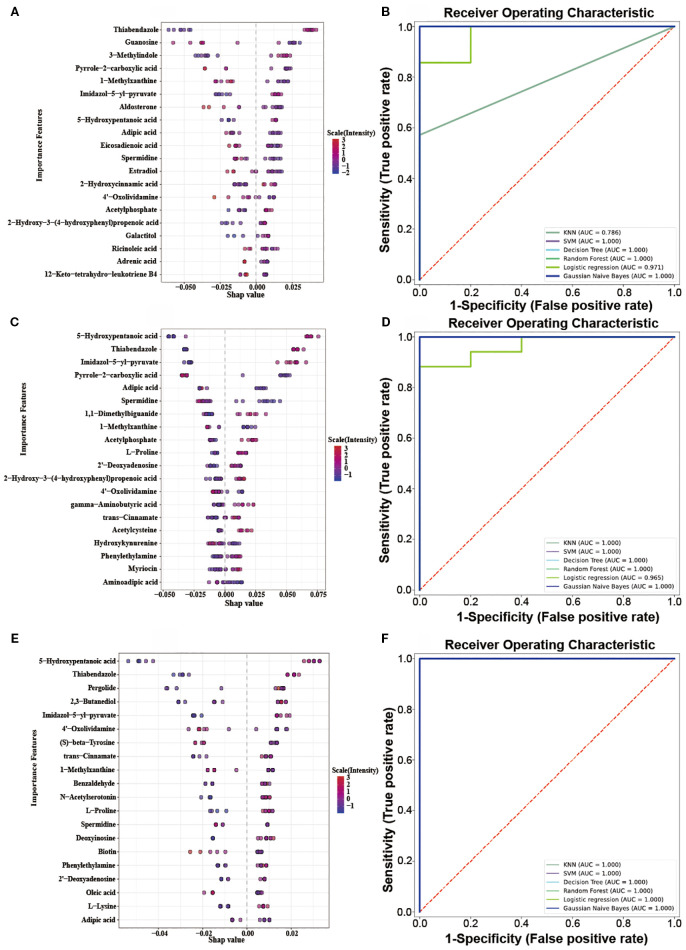
**(A, C, E)**: Top 20 DEMs Bee Swarm Plot in the Level 1vs Level 0, Level 2vs Level 0, Level 3vs Level 0; **(B, D, F)**: Multi-model ROC predictive curve in the Level 1vs Level 0, Level 2vs Level 0, Level 3vs Level 0.

SHAP analysis, grounded in the principles of game theory and local explanations, falls under the category of established *post hoc* interpretive methods. This approach enables the computation of Shapley values, which in turn are employed to quantify the individual contributions of each feature. Versatile and adaptable, SHAP is compatible with a wide range of machine learning algorithms. In our research, we applied SHAP analysis to a set of DEMs in different OIPN groups, using a Random Forest model to ascertain their Shapley values, which serve as a measure of their significance. As depicted in (**Figures 5A, C, E**), these Shapley values are presented for each sample. The importance of the metabolites is ranked based on the absolute average Shapley value, which is then used to normalize the quantitative data for each metabolite within the samples. This normalization helps illustrate how the importance of each feature data point influences the model’s outcomes. The vertical axis in the figure lists the top 20 metabolites with shapely values that exhibit differential effects, while the horizontal axis displays the Shapley scores predicted by the Random Forest model for the test set samples. These scores represent the contribution of each metabolite to the classification prediction of the sample. The magnitude of these scores indicates the relative contribution of the metabolite to the classification results, with higher absolute values suggesting greater importance. The color gradient in the visualization corresponds to the standardized (scaled) characteristic values (quantitative metabolite values) across different samples, with red denoting higher values and blue indicating lower values.

**Figure 6:**
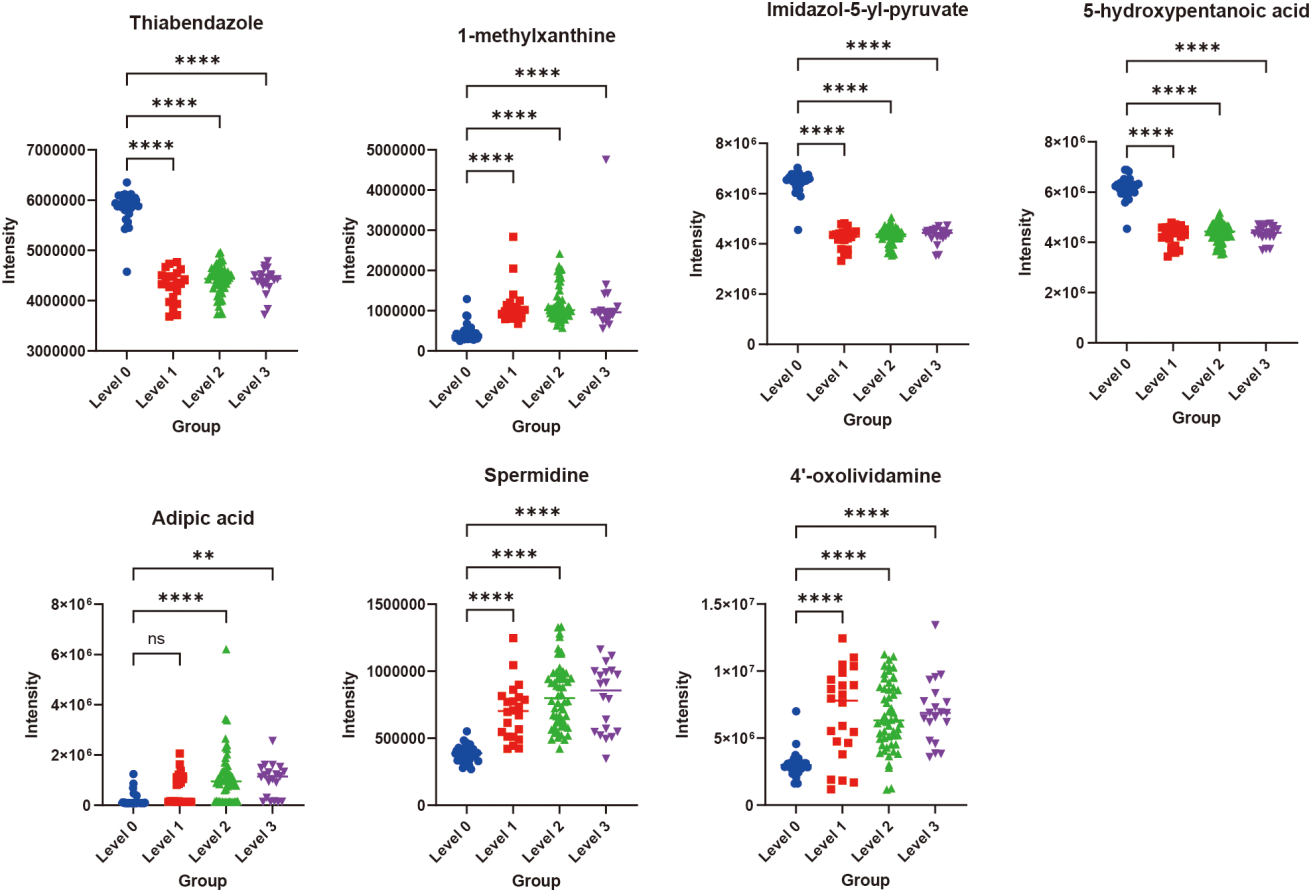
The relative contents of the seven important biomarkers in Level 0, Level 1, Level 2, and Level 3. **p<0.01, ****p<0.0001, ns indicated that there was no significant difference between two groups.

The ROC (Receiver Operating Characteristic) curve can reveal the ability of a machine learning classifier to identify samples at a certain threshold (16). The closer the ROC curve is to the upper-left corner, the higher the TPR (True Positive Rate) and the lower the FPR (False Positive Rate) in the model, indicating higher sensitivity and lower misjudgment rate, and thus better performance of the diagnostic method. The closer the area under the ROC curve is to 1, the better the model’s sensitivity and specificity indicators, and the more ideal the evaluation indicators are, indicating that the selected biomarkers have excellent classification ability and effect. The top 20 DEMs in terms of contribution from SHAP analysis were selected (**Supplementary Table S2**), and classification prediction verification was carried out through six machine learning algorithms, including K-Nearest Neighbor, Random Forest, Support Vector Machine, Gaussian Naive Bayes, Logistic Regression, and Decision Tree. It was found that in the comparisons of Level 1 vs Level 0, Level 2 vs Level 0, and Level 3 vs Level 0, the AUC (Area Under the Curve) values were all relatively high (all AUC values nearly 1) (**Figures 5B, D, F**). These results indicated that the 20 metabolites selected through SHAP analysis in these three groups of analyses had high classification value. It also suggested that the model’s predictive results were not accidental and possess a certain degree of robustness. In the three groups, the relative abundance profiles of seven biomarkers were consistent between subjects with and without OIPN. Notably, as shown in **Figure 6**, except for adipic acid, six biomarkers—thiabendazole, 1-methylxanthine, imidazol-5-yl-pyruvate, 5-hydroxypentanoic acid, spermidine, and 4’-oxolividamine exhibited statistically significant differences (p < 0.0001) when comparing Level 0 (non-OIPN) with Levels 1, 2, and 3 (graded severity of OIPN), highlighting their potential as discriminative markers for distinguishing the presence from the absence of OIPN. These findings suggest metabolic perturbations associated with the development of OIPN, these six metabolites were identified as key differential biomarkers that distinguish patients with OIPN from those without OIPN manifestations, and their distinct metabolic profiles demonstrate significant discriminatory potential in characterizing the pathophysiology of OIPN.

In contrast, among the Level 2 vs Level 1, Level 3 vs Level 1, and Level 3 vs Level 2 comparison groups, the top 20 differential metabolites with the highest contributions were identified through SHAP analysis (**Supplementary Table S3**). Subsequent validation using six machine learning models revealed AUC values ranging between 0.696–0.804, 0.607–0.762, and 0.549–0.843 for these groups, respectively (**Supplementary Figure S5**). These results indicated that these DEMs cannot effectively predict the differences between Level 1, Level 2, and Level 3 (17). This may be attributed to the relatively small sample size in our experiment, and the fact that patients were graded for OIPN based on their sensations, which might have led to the failure in identifying clear differentiating compounds.

### 3.5 Association of six biomarkers with clinical characteristics

**Figure 7:**
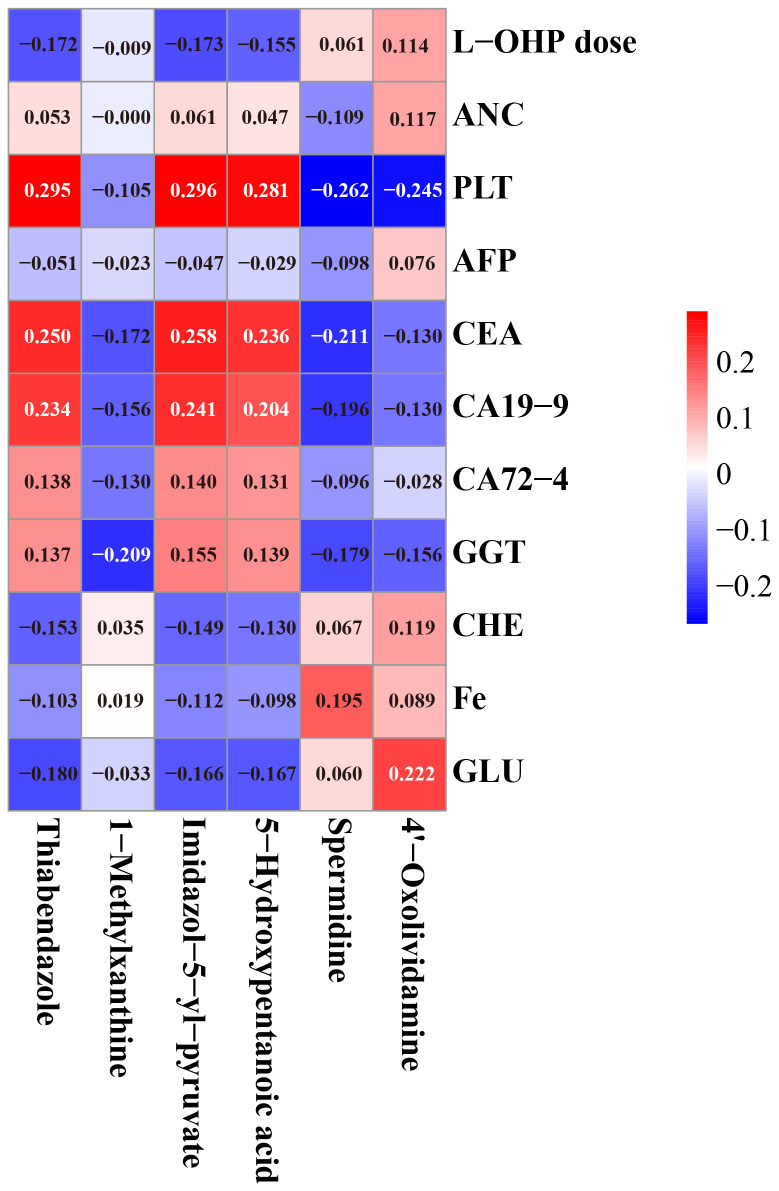
Person correlation analysis between six important biomarkers and clinical features in OIPN (red: positive; blue: negative; higher absolute values indicate stronger correlations).

The results of Pearson correlation analysis between six important biomarkers (including spermidine, thiabendazole, 1-methylxanthine, imidazol-5-yl-pyruvate, 5-hydroxypentanoic acid, and 4’-oxolividamine) and clinical features related to OIPN (including L-OHP dose, ANC, PLT, AFP, CEA, CA19-9, CA72-4, GGT, CHE, Fe, GLU) were shown in **Figure 7**. Red indicates a positive correlation and blue indicates a negative correlation. The absolute value of the correlation coefficient reflects the strength of the correlation (the larger the absolute value, the stronger the correlation). Specifically, the correlations between various biomarkers and clinical features showed different trends. For example, thiabendazole had a relatively certain positive correlation with PLT (correlation coefficient 0.296), and 1-methylxanthine also had a certain positive correlation with PLT (0.295); CEA had a certain positive correlation with Imidazol-5-yl-pyruvate (0.258); CA19–9 had a certain positive correlation with Imidazol-5-yl-pyruvate (0.241); Spermidine had a certain positive correlation with GLU (0.222); However, there were weak negative correlations between some indicators, such as the correlation coefficient between PLT and spermidine was -0.262, the correlation coefficient between CEA and spermidine was -0.211, and the correlation coefficient between GGT and 1-methylxanthine was -0.209. Overall, the absolute values of most correlation coefficients were small, suggesting that the association between these biomarkers and the listed clinical features is mainly weak, but this analysis provides basic data for exploring the potential clinical association of biomarkers in OIPN.

## 4 Discussion

OIPN emerges as a frequent side-effect among colorectal cancer patients. Early detection and management are essential to minimize the risk of discontinuing OIPN, enhance treatment adherence, and ultimately improve the prognosis for OIPN patients undergoing OIPN therapy. In this research, untargeted metabolomics was used to investigate OIPN in colorectal cancer patients. We found that there were significant differences in plasma metabolic profiles in different stages of OIPN. ABC transporters, central carbon metabolism in cancer, amino acid metabolism, and linoleic acid metabolism were significantly affected during the onset of OIPN. SHAP-guided random forest algorithms and six machine learning algorithms further validated thiabendazole, 1-methylxanthine, imidazol-5-yl-pyruvate, 5-hydroxypentanoic acid, spermidine, and 4’-oxolividamine which were associated with OIPN patients (Level 1-3) from non-OIPN controls (Level 0) (**Figures 5**, **6**). However, differentiation between intermediate OIPN grades (Level 1 vs 2, Level 1 vs 3, Level 2 vs 3) yielded lower predictive accuracy (AUC: 0.549–0.843) (**Supplementary Figure S5**). These metabolic features may provide useful clues for future mechanism exploration and identification of therapeutic targets of OIPN.

Previous study has indicated that the incidence of OIPN is dose-dependent, when the cumulative dose reaches 780–850 mg/m^2^, 15% of patients may experience symptoms such as persistent numbness; when the cumulative dose reaches 1170 mg/m^2^, the incidence rate is 50%, and when the cumulative dose reaches 1560 mg/m^2^, the incidence rate is 75% (18). Aligned with prior researches, we found that the doses of L-OHP used by patients with Level 2 and Level 3 OIPN were significantly higher than those without OIPN, indicating that the cumulative dose of L-OHP chemotherapy plays an important role in OIPN. At the same time, our findings implied that the occurrence of OIPN may be associated with the progression of the disease and the patients’ tumor markers (CEA, CA19-9, CA72-4), as well as immune response and inflammation (ANC, PLT), and metabolic and liver function abnormalities (GGT and UA) (**Table 1**).

Our findings revealed that patients with OIPN exhibited significantly elevated serum levels of 1-methylxanthine (1-MX), suggesting its potential role as a biomarker for nerve injury (19). Key mechanistic insights include: Oxaliplatin directly binds to voltage-gated sodium channels (Nav1.6/1.8), inducing neuronal hyperexcitability and aberrant action potentials (20). As an adenosine A_2_A receptor antagonist, 1-MX may exacerbate sodium channel dysfunction by inhibiting adenosine-mediated neuroprotective effects. This synergy could explain the higher incidence of acute cold allodynia and chronic sensory dysfunction in patients with elevated 1-MX levels. Oxaliplatin triggers mitochondrial oxidative stress (mtROS accumulation) and DNA damage, central mechanisms underlying its chronic neurotoxicity (21). 1-MX amplifies these effects by inhibiting phosphodiesterase (PDE), elevating intracellular cAMP levels, and activating the PKA pathway, thereby promoting mitochondrial permeability transition pore (mPTP) opening and enhancing apoptotic signaling. Furthermore, oxaliplatin may disrupt purine metabolic enzymes, leading to abnormal 1-MX accumulation (22). This study demonstrates a positive correlation between serum spermidine levels and the risk of OIPN. Spermidine synergistically exacerbates neuronal hyperexcitability by enhancing TRPV1 and Nav1.8 channel activity in conjunction with oxaliplatin (23). Experimental data reveal that spermidine significantly increases calcium oscillation frequency in dorsal root ganglion neurons. Single-cell sequencing confirms elevated expression of inflammatory cytokines (IL-6, TNF-α) in peripheral blood monocytes of OIPN patients, which correlates positively with serum spermidine levels (24). Additionally, spermidine may impair the clearance efficiency of oxaliplatin metabolites, leading to neurotoxic substance accumulation and accelerated axonal transport dysfunction. In colorectal cancer patients treated with oxaliplatin, individuals who developed OIPN exhibited significantly higher serum levels of 4’-oxolividamine compared to those without neurotoxicity, with its concentration positively correlated to OIPN severity in our study. 4’-oxolividamine, an oxidized metabolite of polyphenolic compounds, is jointly catalyzed by gut microbiota and hepatic CYP450 enzyme systems (25). Its α,β-unsaturated ketone structure enables covalent modification of cellular proteins, thereby inhibiting the Nrf2/ARE antioxidant pathway and leading to reduced ROS scavenging capacity. Oxaliplatin accumulates in the dorsal root ganglia (DRG), inhibits mitochondrial electron transport chain complexes I/III, and induces mitochondrial DNA damage and reactive oxygen species (ROS) overproduction (26). Consequently, elevated 4’-oxolividamine in OIPN patients may exacerbate oxaliplatin-induced oxidative stress, creating a vicious cycle of “ROS-mitochondrial damage-neuronal apoptosis”.

This study found that the level of Imidazol-5-yl-Pyruvate (I5P) is negatively correlated with OIPN. As an intermediate in histidine metabolism, I5P may enhance mitochondrial function by regulating pyruvate metabolism (27, 28). I5P inhibits the activity of lysosomal cathepsin Cathepsin L, blocks the degradation of IκB-α, thereby reducing the release of NF-κB-mediated inflammatory factors (such as IL-6 and TNF-α) and alleviating neuroinflammation (29). The levels of inflammatory factors in OIPN patients are significantly higher than those in non-OIPN patients. I5P may alleviate the severity of OIPN by reducing the release of inflammatory factors. Our study revealed a significant inverse correlation between serum levels of 5-hydroxypentanoic acid (5-HPA) and the incidence of OIPN. 5-HPA, a hydroxylated fatty acid derivative, may participate in mitochondrial energy metabolism (30). Previous studies have shown that intermediates of fatty acid oxidation (acetyl-CoA and ketone bodies) can mitigate oxidative stress by enhancing ATP synthesis and scavenging ROS (31, 32). Oxaliplatin is known to impair mitochondrial complex I/II activity, leading to ROS accumulation and subsequent axonal damage (33, 34). We hypothesize that higher 5-HPA levels may compensate for this deficit by supporting alternative energy pathways or directly neutralizing ROS. This is supported by the structural similarity of 5-HPA to γ-hydroxybutyrate (GHB), a neuroprotective metabolite shown to reduce ROS in dorsal root ganglia neurons. Our study revealed a significant negative correlation between serum thiabendazole levels and the incidence of OIPN. Oxaliplatin induces axonal damage in DRG neurons by inhibiting mitochondrial complexes I/II activity, leading to excessive accumulation of ROS (35). Notably, thiabendazole, as a benzimidazole compound, possesses strong electron-donating capabilities through its benzene and imidazole moieties, potentially exerting antioxidant effects by directly neutralizing hydroxyl radicals (·OH) or superoxide anions (O2^−^). Furthermore, oxaliplatin disrupts gut microbiota homeostasis (36), promoting pathobionts (e.g., Escherichia coli) to release lipopolysaccharide (LPS), which exacerbates neuroinflammation via the TLR4/NF-κB pathway. Interestingly, thiabendazole, as a broad-spectrum antiparasitic agent (37), may reduce LPS leakage by suppressing the overproliferation of specific microbiota components.

This study and our previously published research (8) both focused on the discovery of metabolomic biomarkers for OIPN, but they exhibit key differences in design and objectives. The prior study successfully identified six stable biomarkers distinguishing OIPN patients from non-OIPN controls based on untargeted metabolomics, including racemethionine, stearic acid, 5-aminopentanoic acid, erythritol, aminoadipic acid, and all-trans-retinoic acid. In contrast, the current work represents the first longitudinal analysis specifically targeting OIPN severity grading (Levels 0-3). It revealed significant associations between OIPN occurrence and cumulative oxaliplatin dose, tumor progression (CEA/CA19-9), and immune-inflammatory indicators (ANC/PLT). Furthermore, employing SHAP-guided machine learning, this study identified six novel biomarkers, including thiabendazole, 1-methylxanthine, imidazol-5-yl-pyruvate, 5-hydroxypentanoic acid, spermidine, and 4’-oxolividamine that demonstrated high accuracy in discriminating the presence of OIPN (Level 0 vs. Levels 1-3, AUC ≈ 1) (**Figure 5**). While pathway-level dysregulation overlapped partially with the previous findings (e.g., disturbances in amino acid metabolism), there was no direct overlap in the specific metabolites identified, potentially reflecting biological differences between OIPN onset and progression stages. Notably, the current study highlighted abnormalities in pathways including ABC transporters and central carbon metabolism in cancer, whereas the prior study emphasized arginine biosynthesis, beta-alanine metabolism, and linoleic acid metabolism. Critically, this study did not identify specific biomarkers capable of effectively differentiating intermediate OIPN severity grades: AUC values were 0.696–0.804 for Level 2 vs. Level 1, 0.607–0.762 for Level 3 vs. Level 1, and 0.549–0.843 for Level 3 vs. Level 2 (**Supplementary Figure S5**). This limitation is likely attributable to the subjective sensory-based grading system, cohort size constraints, and the continuous nature of metabolic changes during OIPN progression. Future validation incorporating objective neurophysiological measures within larger cohorts is warranted.

Regarding the possible reasons for the low predictive accuracy of the six identified metabolites among intermediate OIPN grades (e.g., Level 1 vs 2, Level 1 vs 3, Level 2 vs 3), the main points are as follows: First, the sample size of this study is relatively limited, especially the uneven distribution of samples across different grades (e.g., only 20 cases in Level 3), which may make it difficult for the model to capture subtle metabolic differences between grades. Second, OIPN grading is based on the NCI-CTCAE V3.0 criteria, mainly relying on physicians’ evaluation of patients’ subjective sensations. Such subjective scoring may have individual differences and ambiguous boundaries, resulting in insufficient objectivity of the grading itself. In addition, as a progressive process, OIPN may exhibit continuous characteristics in metabolic changes, and the differences in metabolite abundance between intermediate grades may be subtle. However, the currently screened biomarkers are more inclined to distinguish the presence or absence of OIPN (Level 0 vs 1-3) and have low sensitivity to such continuous and subtle grading differences. Finally, metabolic disorders between intermediate grades may involve more complex pathway interactions, and relying solely on the six metabolites may not fully reflect the biological differences between grades. It is necessary to combine multi-dimensional indicators (such as neuroelectrophysiological parameters) to further optimize the model.

## 5 Conclusions

In this study, untargeted metabolomics coupled with SHAP-guided random forest algorithms, and machine-learning were employed to identify differentially expressed metabolites associated with OIPN in colorectal cancer patients. Our results suggest that L-OHP doses, tumor progression, immune response and inflammation may underlie OIPN. We speculate thiabendazole, 1-methylxanthine, imidazol-5-yl-pyruvate, 5-hydroxypentanoic acid, spermidine, and 4’-oxolividamine show significant promise in understanding the occurrence of OIPN. The metabolite signature discovered may provide a foundation for the management of OIPN. However, the further researches, including larger cohort studies and in-depth investigations into underlying mechanisms, are necessary to validate these differential metabolites and confirm abnormalities in metabolomic pathways.

## Data Availability

The original contributions presented in the study are included in the article/**Supplementary Material**. Further inquiries can be directed to the corresponding authors.
