# Supplementary figures and images for: Metabolome profiling by untargeted metabolomics and biomarker panel selection using machine-learning for patients in different stages of peripheral neuropathy induced by oxaliplatin
Source: Front Oncol. 2025 Sep 19;15:1617207. doi: 10.3389/fonc.2025.1617207 (PMC12491010; doi:10.3389/fonc.2025.1617207)

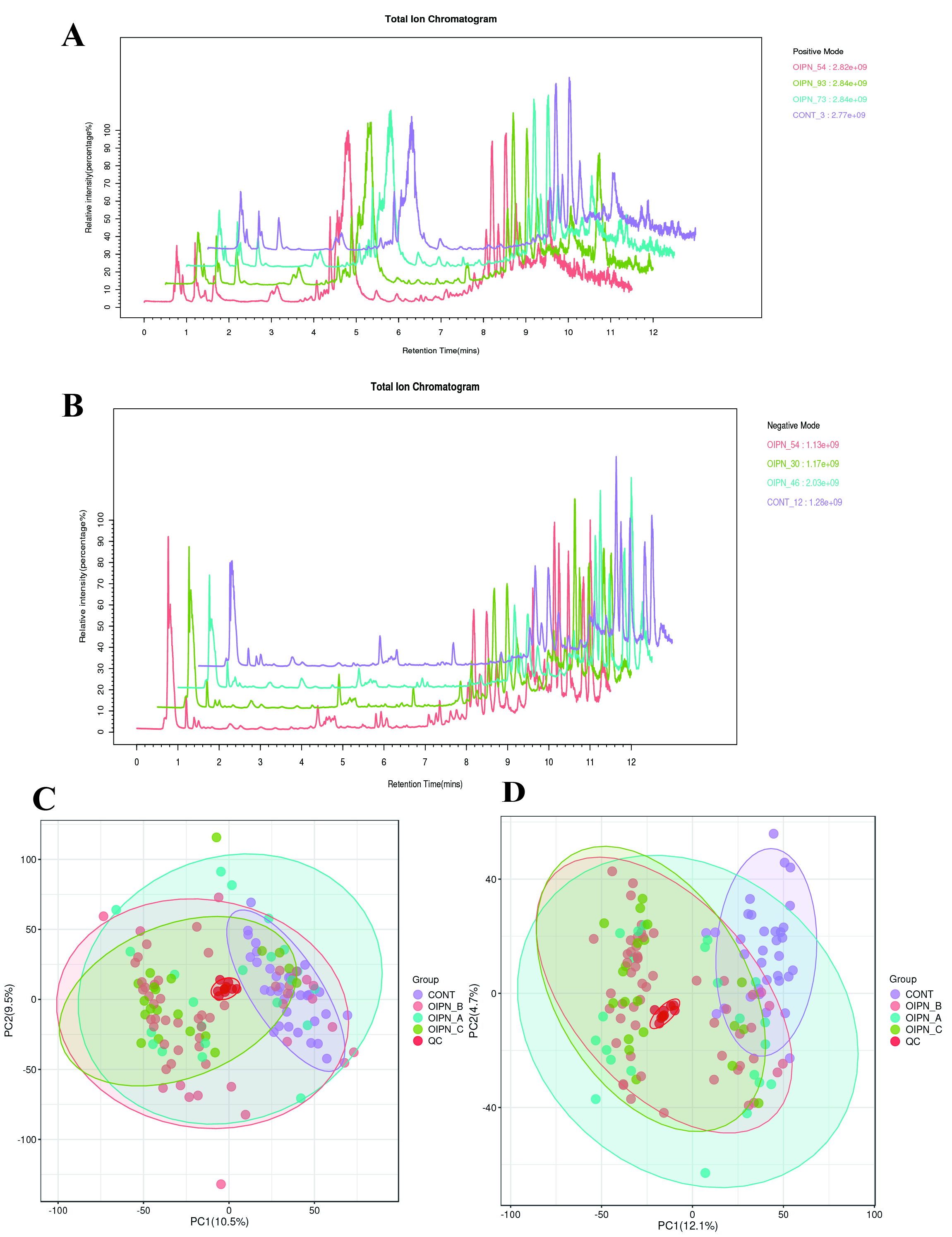

Supplement: Supplementary file 1 [file Image1.tif]

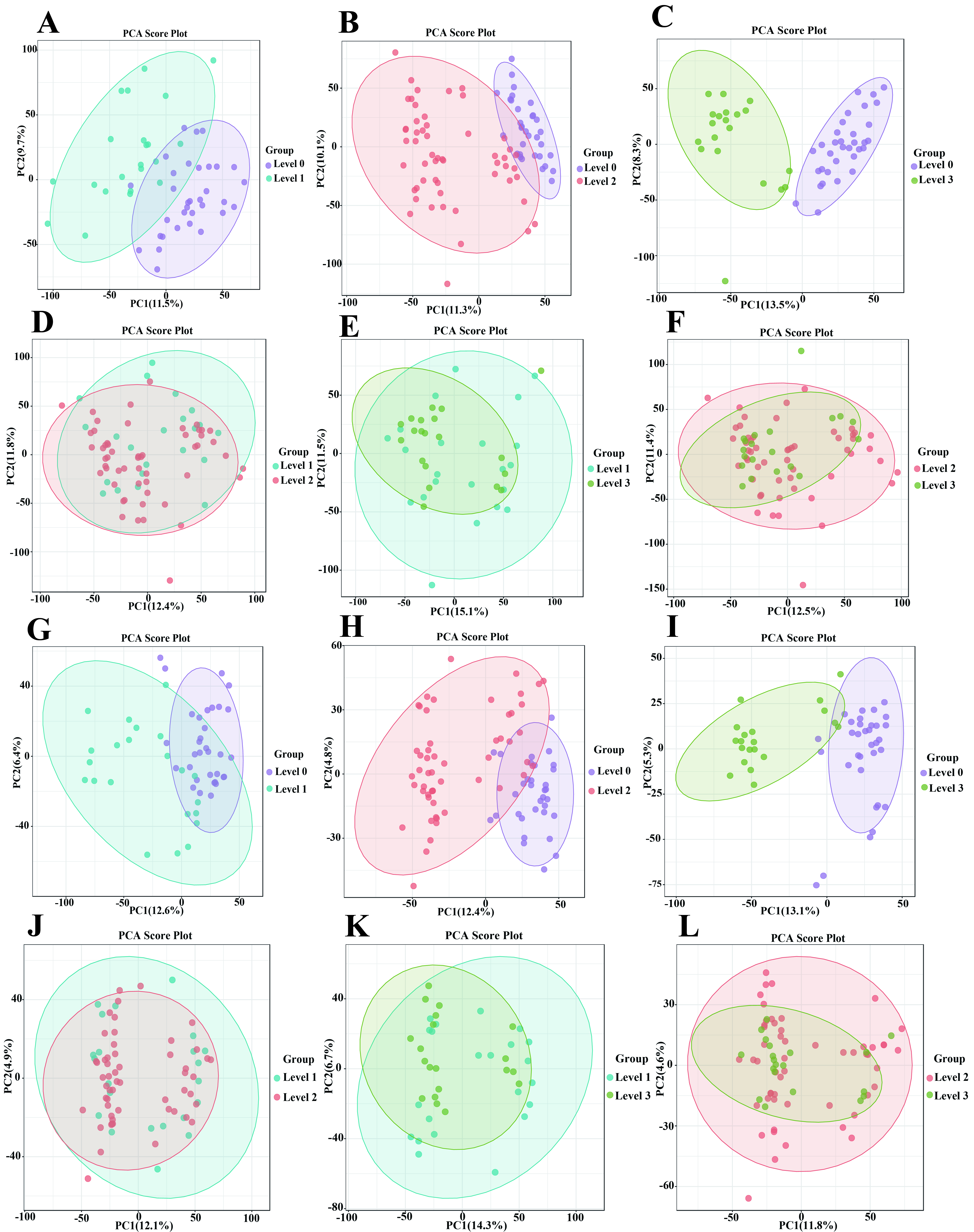

Supplement: Supplementary file 2 [file Image2.tiff]

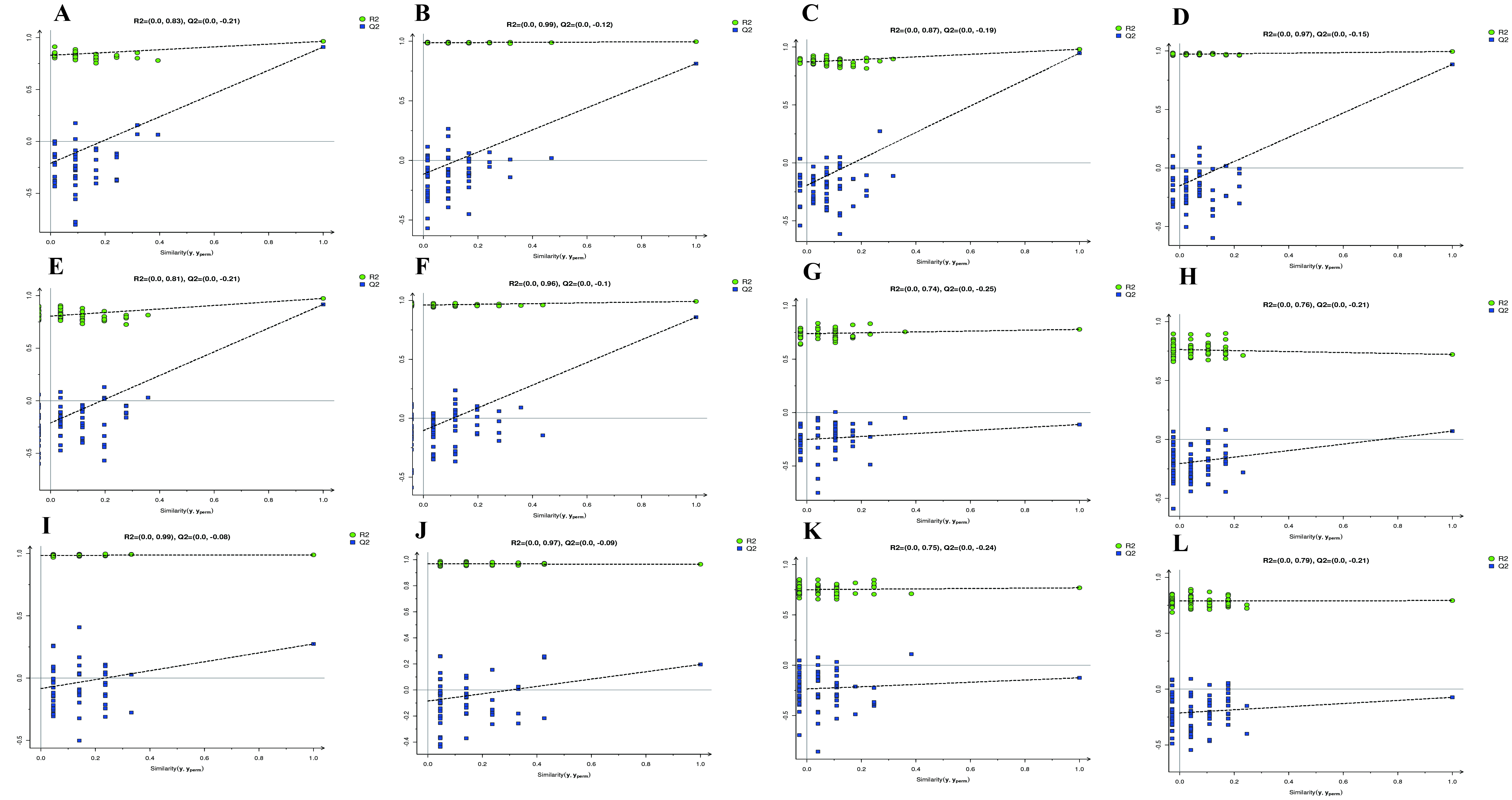

Supplement: Supplementary file 3 [file Image3.tiff]

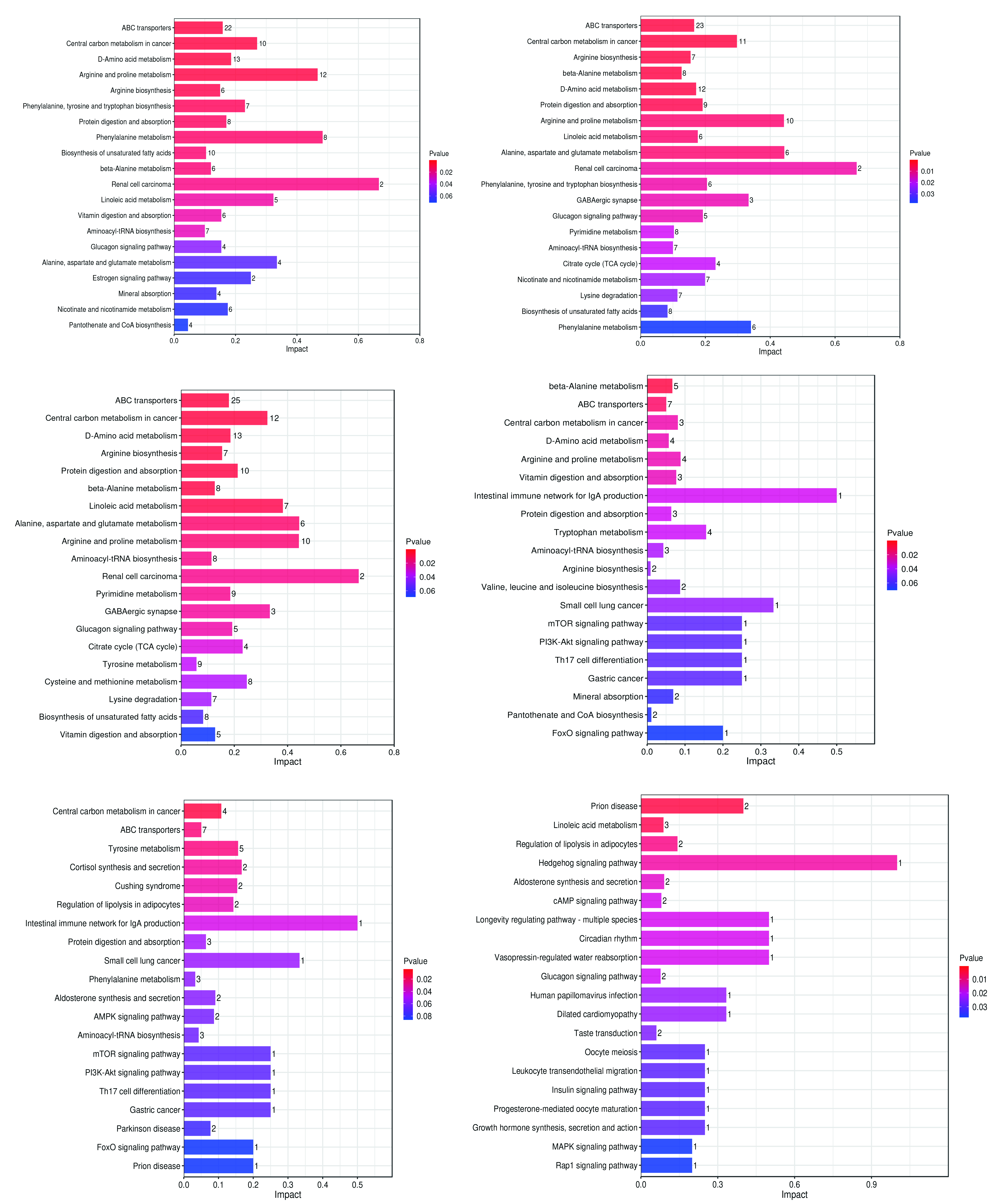

Supplement: Supplementary file 4 [file Image4.tiff]

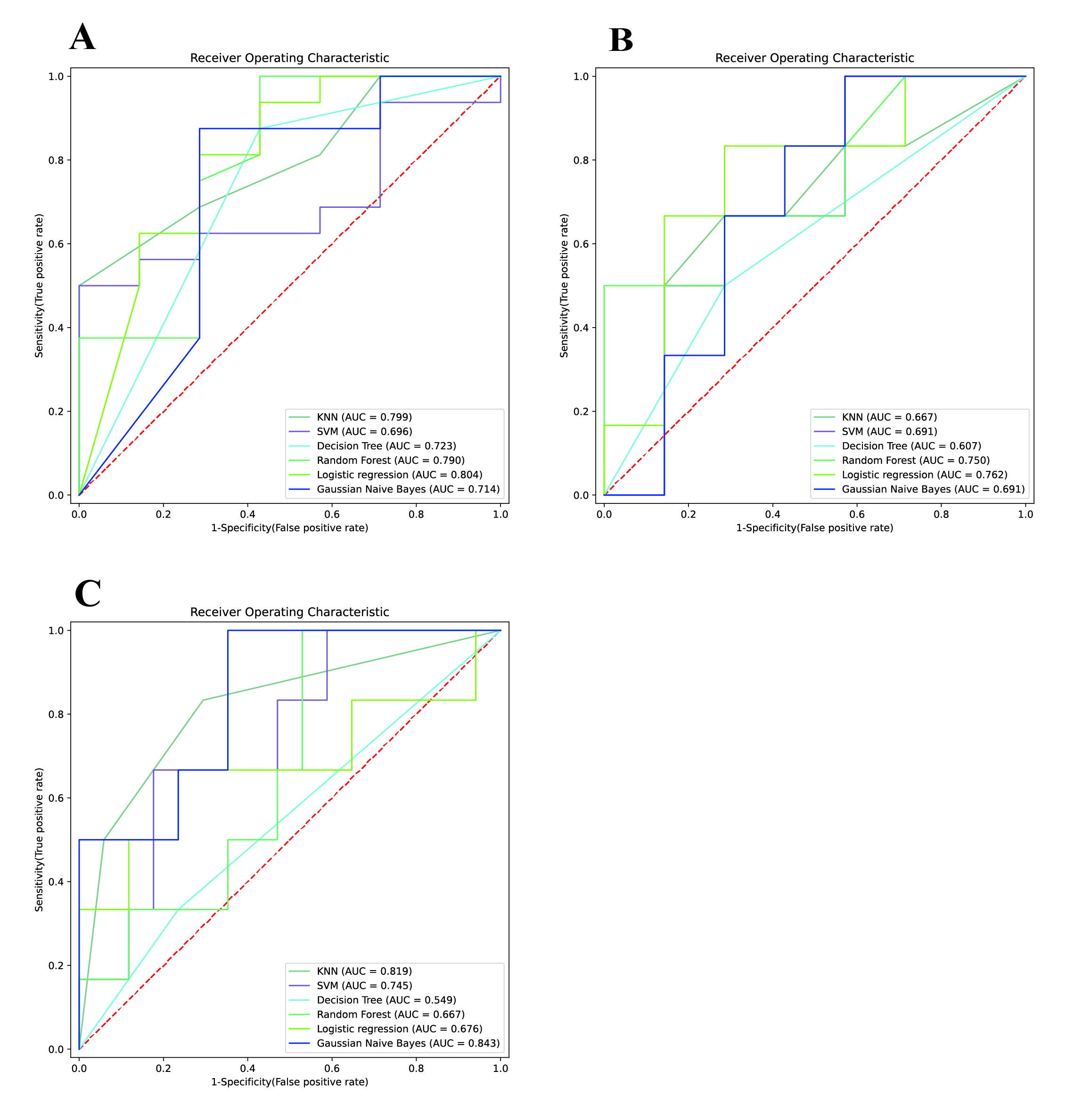

Supplement: Supplementary file 5 [file Image5.tif]
